# Joint association of meal frequency and diet quality with metabolic syndrome in Iranian adults

**DOI:** 10.1186/s40795-022-00507-w

**Published:** 2022-02-07

**Authors:** Neda Azizi, Sakineh Shab-Bidar, Elham Bazshahi, Azadeh Lesani, Mohammad Hassan Javanbakht, Kurosh Djafarian

**Affiliations:** 1 Department of Clinical Nutrition, School of Nutritional Sciences and Dietetics, Tehran University of Medical Sciences (TUMS), Tehran, Iran; 2 Department of Community Nutrition, School of Nutritional Sciences and Dietetics, Tehran University of Medical Sciences (TUMS), Tehran, Iran; 3 Department of Cellular and Molecular Nutrition, School of Nutritional Sciences and Dietetics, Tehran University of Medical Sciences (TUMS), Tehran, Iran

**Keywords:** Metabolic syndrome, Diet quality, Meal frequency, Meal timing, Snack frequency

## Abstract

**Background:**

Metabolic syndrome (MetS) is a common complication that has been shown in various studies to be related to the frequency and timing of eating. We aimed to evaluate the relationship between meal timing and frequency with diet quality and prevalence of MetS.

**Study design:**

Cross-sectional.

**Methods:**

We analyzed data from 850 adults (20 to 59 years) and divided the participants into different categories in terms of frequency of eating occasions (EO) (5 ≥ , 6–7 and 7 <), meal (2 ≥ and 3) and snack (2 ≥ , 3 and 4 ≤) in a day. Daily food consumption was assessed using the structured three 24-h recalls. The quality of diet we calculated using the food quality score (FQS). Metabolic syndrome was defined based on the guidelines of the national cholesterol education program adult treatment panel III (ATP III). The covariates-adjusted relationships between exposures and outcomes were investigated using a logistic regression test and two-way ANOVA.

**Results:**

The overall prevalence of MetS in participants was 34.2%. The average FQS was 28.0. Increased frequency of EOs and snacks was related to the higher prevalence of MetS ((OR, 1.72; 95% CI, 1.24, 2.37; *P* < 0.01) and (OR, 1.34; 95% CI, 1.07, 1.68; P, 0.01), respectively). The adjusted mean of FQS was not significantly different between the EO as well as meals and snack categories. The joint association of EO frequency and snack frequency with diet quality showed a higher chance of having MetS ( (OR, 2.36; 95% CI, 1.19, 4.66; P, 0.01 and (OR, 1.68; 95% CI, 1.06, 2.68; P,0.02), respectively). Also, we observed a higher mean of high density level cholesterol in people with the highest FQS and lowest EO frequency (P,0.02).

**Conclusion:**

Our findings suggest that the EO and snack frequency may be associated with the higher chance of MetS. We also found when the frequency of EO increases, the beneficial associations of the diet quality were overshadowed. To confirm our findings, well designed randomised clinical trials are needed.

## Introduction

Simultaneous occurrence of cardiovascular risk factors is known as metabolic syndrome (MetS), which increases the risk of heart disease and diabetes and other diseases such as bone and joint problems, cancers and fatty liver [1]. This syndrome means the presence of at least three of the following: 1) blood pressure ≥ 130/85 mm Hg 2) fasting blood sugar (FBS) > 100 mg/dl 3) serum triglycerides (TG) ≥ 150 mg/dl, 4) serum high-density lipoprotein cholesterol (HDL) less than 50 mg/dl in women and less than 40 mg/dl in men 5) waist circumference (WC) more than 102 cm in men and more than 88 cm in women [2]. The prevalence of this syndrome in Iran is estimated to be between 20 and 35.8% in 2017, which is higher than in the united states (US) (22.9%) and other western countries (Italy 22% in men and 18%

in women) [1]. The causes of this syndrome include insulin resistance, obesity, especially abdominal, genetic and lack of physical activity, as well as nutritional factors [3–7]. Accumulating evidence suggests that diet composition and dietary patterns are associated with chronic disease [8–12]. All dietary guidelines developed to prevent chronic disease also were based on dietary patterns and composition without considering eating habits. Eating habits including time and frequency of eating per day has been recently investigated with the risk of chronic disease [13–15].

Some evidence reported that increasing the frequency of meals reduces the risk of MetS and its components [16, 17]. However, there is a study that shows higher intake of snacks increase MetS, especially if it contains junk foods [18]. Another study found that eating in the morning and limiting it after 9 PM helps to reduce the odds of MetS [19]. In one study, high frequency and improper timing of food consumption were associated with circadian rhythms disturbances, followed by a disturbance in nutrients metabolism and hormones secretion, and a disturbance in the body's physiological clock, impaired glucose tolerance and insulin sensitivity [20]. In addition to hormonal reasons and circadian rhythms, one of the mechanisms of the effect of time and frequency of meals on MetS can be their relationship with the diet quality. A study found that people whose breakfast was their biggest meal had a higher quality of diet because it was high in fiber and nutritious foods [21]. Another study found that increasing the frequency of main meals was associated with increased intake of vegetables, grains, and fiber, while increased snacks were associated with reduced protein, increased fat, reduced vegetables, grains, and dietary fiber [22].

Results of previous studies regrading association of mealtiming and diet quality with MetS are inconclusive. In addition, no similar study has been conducted in Iran. Therefore the current study aimed to investigate the association of meals and snacks timing and the diet quality with MetS in a sample of Iranian adults.

## Methods

### Population sampling and study design

In this cross-sectional study, a total of 850 men and women aged 20 to 59 years were recruited from an apparently healthy population at health centres of Tehran, the capital city of Iran, using the cluster sampling method. Inclusion criteria were: 1) age group 20–59 years, 2) willingness to participate in the study, 3) apparently healthy adults who did not report any previous diagnosis of chronic diseases such as diabetes, cardiovascular diseases and chronic kidney, lung and liver diseases by a physician, 4) both sexes, and 5) living in Tehran. Exclusion criteria were dietary changes due to a specific or acute illness (cancer, fractures, etc.), pregnancy and lactation. These samples were randomly selected from 25 selected health centers in 5 geographical areas of Tehran and these 25 health centers were randomly selected from 5 regions of Tehran. The collection of samples was carried out with the coordination of the Tehran municipality health department and with the cooperation of health centers in Tehran. The sampling process took 6 months. Information such as age, sex, education, marriage, job and smoking status were collected by the demographic questionnaire.

### Dietary assessment and meal timing

Daily food consumption was assessed using the structured three 24-h recalls of each participant on non-consecutive days (Monday to Sunday). The first 24-h recall was obtained through an interview, and the other 2 recalls were recorded by telephone during two non-consecutive random days. Collecting data using 24-h recall was done using a standard five-step method designed by the united states department of agriculture (USDA) [23]. Meals were known as occasions where large amounts of foods were consumed or were standardized based on time of consumption [24, 25] to contain no more than one breakfast, lunch, and dinner, but allowing for multiple snacks. Breakfast was defined as an eating occasion where a large amount of food or energy was consumed between 5:00 to 11:00 and lunch, if it was consumed between 11:00 and 16:00, then diner was defined as the main meal when was eaten between 16:00 to 23:00 based on prior studies[26]. Participants who consumed the meal on 2-day 24hDRs were used to define meal timing. Participants nutrient intake was analyzed by Nutritionist IV software.

### Frequency and time of food intake

Eating occasions (EO) usually included breakfast, lunch, dinner and snacks. EO was coded for breakfast if was consumed before 11 A.M, as lunch, if was consumed between 11:00 A.M and 4:00 P.M and as dinner, if was consumed between 4:00 P.M to 11:00 P.M. When two or more EOs were reported at the same meal, 59 min apart, they were considered a meal and using the average time of their consumption, mealtime was determined. Otherwise, EOs were coded with more energy content as one meal and other EOs as snacks. For example, EOs that were eaten until 11 A.M if eaten more than 59 min apart, EOs with more energy content were considered as breakfast and other EOs with a time interval of 15 min were considered as snacks. Therefore, people were divided into 2 groups in terms of the number of meals in a day. People who have the number of meals equal to or less than 2 and people who have 3 meals in a day. Also, in terms of the number of snacks in a day, they were divided into three groups: 1) people who have the number of snacks equal to or less than 2, 2) people who have 3 snacks, and 3) people who have 4 ≤ snacks in day. We also divided people into 3 categories in terms of the number of EO in day: 1) people who have the number of EOs equal to or less than 5, 2) people who have 6–7 EOs, and 3) people who have 7 < EOs in a day. In this study, the time of three meals of breakfast, lunch and dinner were also extracted from three food recalls. Meal timing was based on the average meal time of three recalls.

### Diet quality

We used the method defined by Fung et al., food quality score (FQS) [27], for scoring diet quality. The components of FQS includes vegetables, fruits, nuts, legumes, dairy, coffee, poultry and fish, potato, sugar, refined grains, red meat, processed meat, solid oil and butter and sweet chocolate. The vegetables, fruits, nuts, legumes, dairy, coffee, poultry and fish were classified as healthy food groups and potato, sugar, refined grains, red meat, processed meat, solid oil and butter and sweet chocolate were classified as unhealthy food groups. We then ranked participant’s intakes into quintiles and assigned scores between 1 and 5 for healthy foods and assigned reversed quintile rankings scores between 5 and 1 for unhealthy foods. After collecting food groups scores for each person, the overall diet score ranged between 14 to 70. A higher diet score indicates a healthier dietary pattern. The FQS was devided based on the median vaue to two groups of (F1 (29-37) and F2 (18-28).

### Assessment of physical activity

The validated version of the international physical activity questionnaire (IPAQ) [28, 29] was used for the assessment of physical activity. This questionnaire measures people's physical activity based on 5 items: 1) physical activity related to the job, 2) physical activity related to transportation, 3) home business, housekeeping and family care, 4) physical activity related to recreation and leisure, and 5) sitting time [30, 31]. Low, medium and high-level physical activity questions were calculated based on the metabolic equivalent of task (MET) for each sport. Based on this, physical activity was divided into 3 categories: 1) no physical activity, 2) those who have energy consumption between 600 to 1500 min -MET and 3) those who have energy consumption above 1500 min -MET.

### Blood pressure, anthropometric and biochemical assessments

People's blood pressure by digital manometer (BC 08, Beurer, Germany) after 10–15 min’ rest was measured while sitting. Blood pressure was measured twice for each person and the average blood pressure was reported for each person. The waist circumference, between the lowest gear and the iliac crest in the exhalation mode, was measured with a tape measure. Of all participants, 5 ml of blood was taken in fasting between 7–10 A.M and was poured into acid-washed test tubes without anticoagulation until after keeping at room temperature for 30 min and after blood clotting would centrifuge at 1500 g for 20 min. The serums were poured into clean microtubules and stored in a freezer at -80 C ° until the experiment. Measurement of serum glucose and lipid profile using enzymatic method, based on colourimetry, using commercial kits (Pars Azmoun, Iran) with the automatic device (Selecta E, Vitalab, Netherland) done. The experiments were performed on the day of sampling.

### Definition of MetS

We used definition of ATP III for MetS. If a person had at least 3 of the following factors, he or she had MetS: blood pressure ≥ 130/85, FBS > 100 mg/dl, TG ≥ 150 mg/dl, and HDL less than 50 mg/dl in women and less than 40 mg/dl in men and WC more than 102 cm in men and more than 88 cm in women [2].

### Statistical methods

Statistical analysis was performed by SPSS software version 16 and p-value less than 0.05 was considered as a significant level. The Kolmogorov–Smirnov test was used to determine the normal distribution of data. The values ​​of food groups of individuals before calculating FQS adjusted to energy intake using residual methods [32]. To represent and compare the basic quantitative characteristics, energy and energy-adjusted macronutrients intake, FQS and the values ​​of the components of the MetS of the participants across different categories of EO, meal and snack, one-way analysis of variance (one-way ANOVA) and analyze of covariance (ANCOVA) to control confounding variables, were used. Also to show qualitative basic characteristics among different groups of EO, meal and snack, we used the Chi-square test. To describe the basic characteristics of people, mean, standard deviation (SD), frequency and percentage were used. Based on previous studies, the analysis adjusted to sex, age, income, education, marital, occupation and smoking status, energy intake, physical activity level. The relationships between the frequency and timing of food consumption and the prevalence of MetS were assessed using a logistic regression test. Data were present as the odds ratio (OR) and 95% confidence interval (CI). The covariates-adjusted relationships between exposures (frequency of EO, meal and snack and diet quality) and outcome (MetS) was investigated using a logistic regression test. To compare adjusted means of MetS components across tertiles of exposures was investigated using analysis of covariance (ANCOVA). Also, to obtain the adjusted odds of MetS across tertiles of meal occasions or diet quality and MetS we used logistic regression analysis. We also used two-way ANOVA to find a joint association of meal occasions and diet quality with MetS. In both tests, the interaction between frequency of EO, meal and snack, mealtime and diet quality on MetS and its components was investigated.

## Results

The basic characteristics of 850 individuals are shown in detail in Table 1. The average age of participants was 42.3 years. Most participants in the study were women (female = 80.6%, male = 19.3%). Most of them were educated (35.1%), married (80.4%) and non-smokers (95.0%) and almost more than half of them were housekeepers. 52.4% of individuals had low physical activity level. Mean age, and the distribution of sex, income, smoking, physical activity, marriage and occupation did not differ significantly between different snack categories as well as EO and meals. But the level of education was significantly different between different categories of EO and in the higher EO categories, the number of educated people was lower (E3 < E2 < E1; P, 0.04. Furthermore, the energy intake significantly was different in EO and snack tertiles and increased across groups of EO (E1 < E2 < E3; *P* < 0.001) and snacks (S1 < S2 < S3; *P* < 0.001). But different categories of meals did not differ significantly in terms of energy intake. Energy-adjusted mean intake of protein, carbohydrates and fat was not different across categories of main meals, snacks and EOs.

**Table 1:** Baseline characteristics of the study population and daily energy and nutrients intakes according to the EO, meal and snack frequency

	**Meal frequency**				**Snack frequency**				**EO frequency**		
	**1–2**	**3**	***P* Value**	** ≤ 2**	**3**	**4 ≤ **	***P* Value**	** ≤ 5**	**6–7**	**7 < **	***P* Value**
**Age (year)**	41.2) 11.2(	42.5 (10.8)	0.15	42.9 (10.8)	42.3 (10.7)	42.0 (11.1)	0.74	41.9 (11.0)	42.1 (10.9)	44.4 (10.9)	0.24
**Sex (%)**											
** M**	39(23.7)	124(18.2)		23(20.1)	71(21.3)	69(17.3)		29(20.2)	124(19.6)	10(14.4)	
** F**	125(76.2)	556(81.7)		91(79.8)	261(78.6)	329(82.6)		114(79.7)	508(80.3)	59(85.5)	
**Education (%)**			0.52				0.60				0.04*
** Illiterate**	9(5.45)	47(6.91)		8(6.95)	18(5.42)	30(7.53)		11(7.63)	35(5.53)	10(14.4)	
** Under diploma**	33(20.0)	165(24.2)		22(19.1)	81(24.3)	95(23.8)		26(18.0)	153(24.2)	19(27.5)	
** Diploma**	63(38.1)	231(33.9)		37(32.1)	121(36.4)	136(34.1)		49(34.0)	224(35.4)	21(30.4)	
** Educated**	60(36.3)	237(34.8)		48(41.7)	112(33.7)	137(34.4)		58(40.2)	220(34.8)	19(27.5)	
**Occupation (%)**			0.24				0.96				0.98
** Employee**	70(42.4)	238(35.0)		41(35.6)	124(37.3)	143(36.0)		54(37.5)	231(33.6)	23(33.3)	
** Housekeeper**	82(49.6)	362(53.3)		63(54.7)	168(50.6)	213(53.6)		75(52.0)	331(52.4)	38(55.0)	
** Retired**	6(3.63)	41(6.03)		6(5.21)	21(6.32)	20(5.03)		7(4.86)	35(5.54)	5(7.24)	
** Unemployed**	7(4.24)	38(5.69)		5(4.34)	19(5.72)	21(5.28)		8(5.55)	34(5.38)	3(4.34)	
** Income(million)**	1.59(1.21)	1.53(1.05)	0.52	1.49(1.06)	1.59(1.12)	1.52(1.05)	0.59	1.45(1.02)	1.57(1.10)	1.50(1.02)	0.47
**Marriage (%)**			0.22				0.97				0.68
** Single**	28(16.9)	87(12.7)		15(13.4)	47(14.1)	53(13.3)		23(15.9)	86(13.6)	6(8.69)	
** Married**	128(77.5)	552(81.1)		92(80.0)	264(79.5)	324(81.4)		111(77.8)	509(80.5)	60(86.9)	
** Divorced**	4(2.42)	8(1.17)		2(1.73)	4(1.20)	6(1.50)		2(1.38)	10(1.58)	0(0.00)	
** Dead spouse**	5(3.03)	33(4.85)		6(5.21)	17(5.12)	15(3.76)		8(5.55)	27(4.27)	3(4.34)	
**Activity score (%)**			0.21				0.16				0.17
** Low**	96(58.1)	346(51.1)		59 (51.7)	184(55.4)	199(50.2)		74(51.7)	337(53.4)	31(44.9)	
** Moderate**	58(35.1)	266(39.2)		49(42.9)	113(34.0)	162(40.9)		62(43.3)	231(36.6)	31(44.9)	
** High**	11(6.66)	65(9.60)		16(5.26)	35(10.5)	35(8.83)		7(4.89)	62(9.84)	7(10.1)	
**Smoking (%)**			0.74				0.16				0.29
** not smoking**	155(93.9)	648(95.2)		107(93.0)	310(93.3)	386(96.9)		135(93.7)	599(94.7)	69(100)	
** quit smoking**	3(1.81)	11(1.61)		2(1.73)	8(2.40)	4(1.00)		2(1.38)	12(1.89)	0(0.00)	
** low smoking**	7(4.24)	21(3.08)		6(5.21)	14(4.21)	8(2.01)		7(4.86)	21(3.32)	0(0.00)	
**WC (cm)**	89.7(12.1)	88.9(11.8)	0.45	87.0(11.0)	89.5(12.1)	89.3(11.8)	0.12	87.2(11.2)	89.1(11.9)	93.0(11.7)	0.01*
**SBP (mmHg)**	117(16.0)	118(16.1)	0.96	115(13.7)	118(16.3)	118(16.5)	0.10	115(14.3)	118(16.4)	122(15.5)	0.02*
**DBP (mmHg)**	79.7(9.62)	78.9(11.6)	0.31	78.4(10.6)	78.7(10.3)	79.5(12.1)	0.52	78.8(10.4)	79.0(11.4)	80.2(11.7)	0.86
**FBS (mg/dl)**	106(32.0)	108(44.7)	0.63	106(33.0)	104(27.1)	112(53.8)	0.02*	105(30.9)	107(42.2)	122(60.6)	0.02*
**HDL (mg/dl)**	48.9(10.4)	50.1(10.2)	0.49	51.1(11.3)	49.1(9.70)	50.2(10.4)	0.32	50.5(11.1)	49.9(9.94)	49.0(11.5)	0.44
**TG (mg/dl)**	143(75.9)	145(79.8)	0.58	132(6.46)	144(4.18)	149(4.19)	0.03*	136(71.9)	145(79.3)	158(89.3)	0.07
**Mets prevalence (%)**	57(34.5)	234(34.4)	1.00	27(23.4)	114(34.3)	150(37.6)	0.01*	35(24.3)	220(34.8)	36(52.1)	< 0.001*
**Energy(kc)**	1660(367)	1680(382)	0.55	1505.(337)	1686(398)	1717(361)	< 0.001*	1523(333)	1707(380)	1706(387)	< 0.001*
**Protein(gr)**	58.7(19.4) (14.1%)^C^	57.7(14.7) (13.7%)^C^	0.15	53.3(15.8) (14.1%)^C^	57.8(14.4) (13.7%)^C^	59.3(16.5) (13.8%)^C^	0.78	53.8(15.5) (14.1%)^C^	58.8(15.6) (13.7%)^C^	58.3(15.8) (13.6%)^C^	0.96
**Carbohydrate(gr)**	238(57.9) (57.3%)^C^	252(117) (60.0%)^C^	0.19	225(152) (59.8%)^C^	251(101) (59.5%)^C^	254(98.5) (59.1%)^C^	0.76	227(138) (59.6%)^C^	254(134) (59.5%)^C^	249(57.9) (58.3%)^C^	0.76
**Fat(gr)**	58.5(35.5) (31.7%)^C^	56.9(30.2) (30.4%)^C^	0.29	53.1(40.0) (31.7%)^C^	57.7(38.3) (30.8%)^C^	58.0(19.8) (30.4%)^C^	0.26	53.0(36.1) (31.3%)^C^	58.0(30.9) (30.5%)^C^	58.9(21.8) (31.0%)^C^	0.43
**FQS**	27.9(3.15)	28.0(3.06)	0.93	27.3(2.91)	28.1(3.06)	28.0(3.12)	0.23	27.3(2.96)	28.1(3.05)	28.2(3.36)	0.19

All values are presented as means (SD) or n (%), *M* male, *F* female, Activity score: Low no physical activity, Moderate 600–1500 MET/min, High 1500 MET/min ≤ , Smoking: Low 5 ≥ cigarettes/day, Relatively low 6–15 cigarettes/day, Middle 15–25 cigarettes/day, High 25 ≤ cigarettes/day, MetS metabolic syndrome, *TG* triglyceride, *FBS* fasting blood sugar, *SBP* systolic blood pressure, *DBP* diastolic blood pressure, *FQS* food quality score, *HDL* high-density lipoprotein, *WC* waist circumference, *EO* eating occasion, ANOVA and ANCOVA tests used for continuous variables, Chi square test used for categorical variables,* is *p* value < 0.05, the protein, carbohydrate and fat intake adjusted to energy intake, the analysis of FQS and MetS adjusted to age, sex, education, occupation, income, marriage, physical activity, smoking, energy intake, C percentage from energy

The average FQS of the participants was 28.0. The mean of FQS in the EO, meal and snack categories are shown in Table 1. As can be seen, the adjusted FQS had no significant difference between the EO as well as meals and snack categories. However before adjusting on the confounding factors include to age, sex, education, occupation, income, marriage, physical activity, smoking, energy intake, EO categories had significantly different FQS ( P,0.02) and among the EO categories, E2 had significantly higher FQS than E1 (P,0.02). The overall prevalence of MetS in participants was 34.2%. The prevalence of MetS significantly varied between different categories of EO (E1 < E2 < E3; *P* < 0.001) and snack (S1 < S2 < S3; P,0.01). The mean of WC (P,0.01) and SBP (P,0.02) significantly increased with the higher EO frequency. Also, the mean of FBS level had a significant increase with higher EO frequency (P,0.02) and snack frequency so that those in the third category of snack had a higher mean of FBS than the S2 (P,0.02). The mean TG level increased significantly with a higher snack frequency (P,0.02).

The results of the logistic regression test in Table 2 showed that there was no significant relationship between breakfast, lunch and dinner time and the prevalence of MetS. Moreover, the increased frequency of EO and snack were associated with higher odds of MetS ((OR, 1.72; 95% CI, 1.24, 2.37; *P* < 0.01) and (OR, 1.34; 95% CI, 1.07, 1.68; P,0.01), respectively). But there was no such relationship between meal categories and odds of MetS.

**Table 2:** Odds ratios and confidence interval for MetS to meal, EO and snack frequency and meal time

		Meal frequency	P for trend	Snack frequency	P for trend	EO frequency	P for trend	B Time	P for trend	L Time	P for trend	D Time	P for trend
MetS	Crude	0.99(0.69,1.42)	0.97	1.31(1.07,1.62)	< 0.01*	1.81(1.34,2.45)	< 0.001*	0.99(0.99,1.00)	0.41	1.00 (0.99,1.00)	0.73	0.99(0.99,1.00)	0.56
	Adjusted	0.90(0.62,1.32)	0.61	1.34(1.07, 1.68)	0.01*	1.72(1.24, 2.37)	< 0.001*	0.99(0.99,1.00)	0.28	1.00 (0.99,1.00)	0.47	0.99(0.99,1.00)	0.54

All values are presented as OR(CI) using logistic regression test, adjusted for age, sex, education, occupation, income, marriage, physical activity, smoking, energy intake, *EO* eating occasion, MetS metabolic syndrome, *B Time* breakfast time, *L Time* lunch time, *D Time* dinner time,a is reference group, * is *p* for trend < 0.05

Table 3 shows the results of interaction and simultaneous increasing the frequency of EO, snack and meal frequency and a higher FQS with MetS. Before controlling confounders, the interaction between the association of frequency of snacks and FQS with MetS was not significant (P,0.06). However, after controlling for covariates, increasing the number of EO frequency (OR, 1.16; 95% CI, 1.03, 1.32; P,0.01) as well as snack frequency (OR, 1.10; 95% CI, 1.00, 0.1.21; P,0.03) with a higher FQS resulted in an increased risk of MetS.

**Table 3:** Relationship between interaction of meal, EO and snack frequency, meal time and FQS with MetS

		Meal frequency* FQS	P trend	Snack frequency*FQS	P trend	EO frequency*FQS	P trend	B Time*FQS	P trend	L Time*FQS	P trend	D Time*FQS	P trend
MetS	Crude	1.03(0.91,1.17)	0.59	1.08(0.99,1.18)	0.06	1.16(1.03,1.30)	0.01*	1.03(0.90,1.17)	0.65	1.02(0.90,1.16)	0.67	0.99(0.87,1.13)	0.98
	Adjusted	1.04(0.91,1.19)	0.51	1.10(1.00, 1.21)	0.03*	1.16(1.03, 1.32)	0.01*	1.06(0.93,1.22)	0.35	1.07 (0.94,1.23)	0.28	1.02(0.89,1.17)	0.73

All values are presented as OR(CI), * is *p* for interaction < 0.05, adjusted for age, sex, education, occupation, income, marriage, physical activity, smoking, energy intake, using logistic regression test, FQS adjusted to energy intake, (29-37) and (18-28) categories of adjusted FQS, (06:00–08:00) and (08:01–10:40) categories of breakfast time, (12:30–13:55) and (14:00–16:00) categories of lunch time, (18:00–20:40) and (20:41–23:00) categories of dinner time, FQS and meal, EO and snack frequency, meal time independent variables, MetS dependent variable

Table 4 also shows the results of interaction between the association of frequency of EO, snack and meal frequency and FQS on MetS components. We observed higher HDL levels in people with high FQS (F1) and low EO frequency (EO frequency ≤ 5), (P,0.02). However, no significant interaction not found for the associations of frequency of snacks and FQS on the components of the MetS. Interaction between effects of EO frequency and FQS on HDL levels before controlling confounders, was significant (P,0.04).

**Table 4:** Interaction between effects of EO, snack and meal frequency, meal time and FQS on MetS components

			FQS*EO frequency	FQS*snack frequency	FQS*Meal frequency	FQS*Breakfast Time	FQS*Lunch Time	FQS*Dinner Time
FBS (mg/dl)	***P* value**	**Crude**	0.14	0.80	0.72	0.84	0.11	0.79
		**Adjusted**	0.15	0.83	0.81	0.86	0.13	0.74
TG (mg/dl)	***P* value**	**Crude**	0.04*	0.21	0.14	0.25	0.24	0.14
		**Adjusted**	0.06	0.24	0.13	0.22	0.26	0.10
WC (cm)	***P* value**	**Crude**	0.76	0.20	0.49	0.33	0.21	0.47
		**Adjusted**	0.70	0.15	0.44	0.36	0.23	0.38
SBP (mmHg)	***P* value**	**Crude**	0.42	0.08	0.58	0.64	0.51	0.35
		**Adjusted**	0.45	0.09	0.56	0.64	0.51	0.38
DBP (mmHg)	***P* value**	**Crude**	0.59	0.47	0.68	0.89	0.70	0.51
		**Adjusted**	0.60	0.48	0.68	0.89	0.70	0.51
HDL (mg/dl)	***P* value**	**Crude**	0.02*	0.10	0.82	0.96	0.26	0.14
		**Adjusted**	0.02*	0.10	0.85	0.98	0.26	0.14

All values are presented as mean (SD),adjusted for age, sex, education, occupation, income, marriage, physical activity, smoking, energy intake, two-way ANOVA used for continuous variables, FQS adjusted to energy intake, (29-37) and (18-28) categories of adjusted FQS, (06:00–08:00) and (08:01–10:40) categories of breakfast time, (12:30–13:55) and (14:00–16:00) categories of lunch time, (18:00–20:40) and (20:41–23:00) categories of dinner time, a is reference group, * is *p* value < 0.05, *TG* triglyceride, *FBS* fasting blood sugar, *SBP* systolic blood pressure, *DBP* diastolic blood pressure, *WC* waist circumference, *HDL* high-density lipoprotein, *FQS* food quality score, *D Time* dinner time, *L Time* lunch time, *B Time* breakfast time

## Discussion

In this cross-sectional study of 850 participants aged 20 to 59 years, no significant relationship was found between the frequency and timing of meals and the prevalence of MetS and its components. The EO and snack frequency, regardless of the diet quality, increased the risk of MetS. When we examined the joint association of EO or snack frequency and diet quality with MetS, we observed that EO and snack frequency rather than diet quality had a significant association with MetS. It means when the frequency of EOs increased, the risk of MetS increased significantly, even in the group of people with high diet quality (F1). We also found a relationship between diet quality and EO frequency only for HDL. But there was no interactive and synergistic correlation of diet quality and both meal and snack frequency and meal timing with MetS components.

To our knowledge, this is the first study that examined these associations between diet quality and eating frequency, frequency of both meals and snacks and meal timing and MetS and its components. It has been suggested that three regular meals consumption per day have beneficial effects on metabolic health and therefore is recommended to the general population, however, the modern westernized lifestyle is characterized by irregular meal patterns such as skipped meals. Although there is limited scientific evidence to support this recommendation. Evidence suggests that research should move beyond specific foods and concentrate on eating habits, including the frequency of EO [33]. Meal patterns with nutrient intakes and diet quality are necessary to determine as markers of the healthiness and variety of the whole diet [34]. Research suggests that different meal and/or snack patterns are related to both nutrient intakes and overall diet quality.

Epidemiological studies on EO frequency have reported an inconsistent association between meal frequency with body weight and adiposity. Some studies showed higher meal frequency is related to lower body weight or obesity [35–37]. While other studies did not show any significant association [38, 39]. Our findings are consistent with previous studies, that showed a direct relationship between the EO frequency and adiposity among adults in the USA [40, 41], and in British adults [42]. In contrast, Jung et al. [16] found that a higher meal frequency was associated with a lower prevalence of MetS in men but not in women. Also in a sample of young Australian adults, a higher frequency of EO was significantly associated with reduced cardiometabolic risk factors in men only [43]. Moreover, Ha et al. [44] indicated that a greater frequency of EO was associated with a lower prevalence of metabolic abnormalities. In addition, a study with 2372 African-American and Caucasian girls (9–19 years), conducted by Ritchie et al. [45] found that lower meal frequency was independently related to greater increases in BMI and WC over 10 years. These contradictory results can be justified by various factors. First, socio-economic and lifestyle variables, which may confound the results. The adjusted covariates were not consistent among studies. Second, under-reporting of energy intake, which is an issue in common observational studies especially when relying on self-reported diet recalls, maybe masked the exact association between eating frequency and metabolic outcomes [46]. Finally, there have been some different approaches in the definition of eating frequency. It is worth noting that meal frequency effects are strictly related to meal timing and macronutrients uptake. However, we failed to show a relationship between meal timing with MetS and its components. The available data about the effects of small, frequent meals compared to gorging large, infrequent meals on isoenergetic conditions [47] provide conflicting results, probably due to the above-mentioned confounding factors.

In the present study, we showed that the higher chance of MetS induces alone with increasing frequency of EO and snacks and decreases with an increasing diet however, the simultaneous effect of the EO and snack frequency with the diet quality showed that the effect of the frequency is more dominant than the diet quality. This dominant effect of frequency rather than the diet quality was also observed for HDL. Evidence suggests that regardless of snack macronutrient composition, consuming snacks especially in a non-hungry state can lead to overeating and, potentially, weight gain [48]. One of the mechanisms to enhance appetite control via which increased meal frequency is by preventing large decreases in plasma glucose between meals. The second mechanism by which more frequent meals has been suggested to reduce hunger is by sustaining higher levels of between-meal insulin concentrations, which would be expected to lead to reduced hunger [49]. However, Ohkawara et al. [50] reported that consuming smaller, more frequent meals may have adverse effects on hunger and fullness. They showed that the insulin area under the curve was significantly lower during 6 M than 3 M. Furthermore, insulin tended to fall to similar concentrations after the consumption of each meal. They suggest that it is unlikely that increasing meal frequency from 3 to 6 M alters the rate of decline in glucose or insulin concentrations between meals in a manner that would influence appetite and food intake patterns. Increasing meal frequency caused more frequent peaks in glucose and insulin with only minor effects on AUC responses.

As we reported, after adjusting for energy, the diet quality does not differ between the different categories of EO, snack and meal frequency. It has been shown that fewer meals in 24 h with large amounts of high-energy food consumed in each meal especially in overweight people, increase overeating and is associated with high total energy intake [51]. In a randomized cross-over study reducing the frequency of meals on health outcomes in normal-weight adults without any changes in overall energy intake [52], improved cardiovascular risk factors and body composition. The mechanisms behind the association of eating frequency on weight management are not clear. It is been reported that reducing the eating frequency may have negative effects on controlling appetite [53]. Another hypothesis is that increasing the eating frequency may increase dietary-induced thermogenesis [54–56]. According to the conflicting results among published studies and lack of enough data in this area, further well-designed studies are required to show the role of meal frequency on body composition and weight changes.

To our knowledge, this is the first observational study to investigate the associations of meal timing and frequency, snack and EO frequency with MetS and its components based on diet quality of Iranian adults. Also, we recruited a proportional amount of participants from the five regions within health centers and a relatively large number of participants. Indeed, Tehran is the Capital of Iran and has a multiethnic population, and in health research in Iran, the population of Tehran are considered as a representative of Iran. The present study had some limitations. First, the cross-sectional design of the study makes it difficult to conclude. Second, the use of 24-h dietary recall data may have been subject to recall bias of self-reported timing and measurement error due to within-subject variations. Third, random measurement errors in the assessment of dietary intake may attenuate the associations between dietary intake and health outcomes. Fourth, our tools for assessing the quality of the diet should have been more accurate. Fifth, we did not examine the effect of skipping meals on the risk of MetS. Sixth, individuals may not have been careful in filling out the questionnaires.

## Conclusion

Our findings suggest that the EO and snack frequency may be associated with a higher chance of MetS. We also found when the frequency of EO increases, the inverse association of the FQS and MetS were overshadowed. Although meal timing and frequency in humans are affected by behavioral factors as well as physiological factors, their efficacy can be used as one of the strategies to improve eating patterns in modern society according to diverse environmental challenges like longer nightlife and ease of access to food.

## Data Availability

The datasets generated during the current study are not publicly available but are available from the corresponding author on reasonable request.

## Abbreviations

MetS: Metabolic syndrome
FBS: Fasting blood sugar
TG: Triglycerides
HDL: High-density lipoprotein cholesterol
WC: Waist circumference
USDA: United states department of agriculture
EO: Eating occasion
FQS: Food quality score
IPAQ: International physical activity questionnaire
MET: Metabolic equivalent of task
one-way ANOVA: One-way analysis of variance
ANCOVA: Analyze of covariance
SE: Standard error
OR: Odds ratio
CI: Confidence interval
